# Adiponectin and adiponectin receptor 1 overexpression enhance inflammatory bowel disease

**DOI:** 10.1186/s12929-018-0419-3

**Published:** 2018-03-14

**Authors:** Yu-Ju Peng, Tang-Long Shen, Yu-Shan Chen, Harry John Mersmann, Bing-Hsien Liu, Shih-Torng Ding

**Affiliations:** 1 Department of Animal Science and Technology, National Taiwan University, No. 50, Ln. 155, Sec. 3, Keelung Rd., Da’an Dist, Taipei City, 10617 Taiwan; 2 Department of Plant Pathology and Microbiology, National Taiwan University, No.1, Sec.4, Roosevelt Road, Taipei, 10617 Taiwan

**Keywords:** Adiponectin, Adiponectin receptor 1, IBD, Il-8, Neutrophils, CXCL1, CXCL2, CXCL5

## Abstract

**Background:**

Adiponectin (ADN) is an adipokine derived from adipocytes. It binds to adiponectin receptor 1 and 2 (AdipoR1 and R2) to exert its function in regulating whole-body energy homeostasis and inflammatory responses. However, the role of ADN-AdipoR1 signaling in intestinal inflammation is controversial, and its role in the regulation of neutrophils is still unclear. Our goal was to clarify the role of AdipoR1 signaling in colitis and the effects on neutrophils.

**Methods:**

We generated porcine AdipoR1 transgenic mice (pAdipoR1 mice) and induced murine colitis using dextran sulfate sodium (DSS) to study the potential role of AdipoR1 in inflammatory bowel disease. We also treated a THP-1 macrophage and a HT-29 colon epithelial cell line with ADN recombinant protein to study the effects of ADN on inflammation.

**Results:**

After inducing murine colitis, pAdipoR1 mice developed more severe symptoms than wild-type (WT) mice. Treatment with ADN increased the expression of pro-inflammatory factors in THP-1 and HT-29 cells. Moreover, we also observed that the expression of cyclooxygenase2 (cox2), neutrophil chemokines (CXCL1, CXCL2 and CXCL5), and the infiltration of neutrophils were increased in the colon of pAdipoR1 mice.

**Conclusions:**

Our study showed that ADN-AdipoR1 signaling exacerbated colonic inflammation through two possible mechanisms. First, ADN-AdipoR1 signaling increased pro-inflammatory factors. Second, AdipoR1 enhanced neutrophil chemokine expression and recruited neutrophils into the colonic tissue to increase inflammation.

**Electronic supplementary material:**

The online version of this article (10.1186/s12929-018-0419-3) contains supplementary material, which is available to authorized users.

## Background

Inflammatory bowel disease (IBD) is a chronic, idiopathic gastrointestinal tract inflammation, including severe forms such as Crohn’s disease and ulcerative colitis [1, 2]. Due to the dramatically increasing incidence worldwide, IBD has become a noticeable health problem over the past decades [3, 4]. In the USA and Europe, there are over 1 million and 2.5 million people with IBD, respectively [1, 5, 6]. Although IBD is not a life-threatening disease, the IBD patients have poor life quality due to loss of body weight, diarrhea, rectal bleeding and abdominal pain [7]. Moreover, robust evidences suggest that IBD patients may have a higher risk to develop colon cancer [8–10].

Adiponectin (ADN) is an adipokine secreted from adipocytes, and various forms of ADN have been found in plasma. The full-length ADN includes 4 regions (a signal peptide, a variable region, a collagen-like fibrous domain and a c1q-like globular domain) and exists in multiple forms (trimer, hexamer and oligomer) [11, 12]. Globular ADN is formed when the collagen-like domain of full-length ADN is cleaved by elastase [13]. ADN can trigger the activation of AMP-activated protein kinase (AMPK), peroxisome proliferator activated receptor alpha (PPARα) and p38 MAP kinase (p38 MAPK) through its receptors, adiponectin receptor 1 (AdipoR1) and 2 (AdipoR2) [14].

It is well known that ADN improves several different diseases, therefore, enhancing ADN signaling might be a potential therapeutic strategy for the metabolism-related diseases [15]. However, the role of ADN and AdipoR1 in chronic inflammatory and autoimmune diseases, especially in IBD, is still controversial [16]. Previous studies support the protective role of ADN and AdipoR1 in chemically-induced murine colitis [17–21]. However, others implicate ADN in exacerbation of murine colitis [22, 23]. Most studies use a similar experimental model (induction of murine colitis by DSS or 2,4,6-trinitrobenzene sulfonic acid in ADN knock-out mice). Despite this, the results are discrepant. The controversial results let us speculate that ADN might not be the only factor related to inflammation in ADN Knock-out mice.

The innate immune system is the first line of defense against invading pathogens. Neutrophils are one of the most abundant and important immune cells in the innate immune system [24]. Neutrophils, as the effector cells of acute and chronic inflammation, play a role in the maintenance of intestinal homeostasis and pathogenesis of IBD [25]. At the early stages of inflammation in patients with IBD, neutrophils infiltrate into the inflamed intestine, clear pathogenic microbes, and resolve inflammation. However, excessive neutrophil infiltration and accumulation in the colonic epithelium causes damage by production of reactive oxygen species (ROS) [25, 26]. Previous studies show that neutrophils play a crucial and dual role in IBD, and either depletion or upregulation of neutrophils can ameliorate murine colitis [26–28]. Hence, the dual role of neutrophils might lead to the controversial results in previous studies [26, 29]. Furthermore, previous studies demonstrate that ADN inhibits neutrophil apoptosis and enhances the expression of neutrophil-attracting chemokines (CXCL1 and CXCL2 in colonic cells; IL-8 in fibroblasts and hepatocytes) [22, 30–32]. Regardless, the relationship between ADN-AdipoR1, neutrophils and IBD is still unclear.

Earlier studies regarding the effects of ADN on intestinal inflammation mostly use ADN knock-out mice or ADN recombinant protein treatment [17–20, 22, 23]. It should be noted that development of ADN as a pharmacological agent is difficult due to its multiple forms and high level (2–20 μg/mL) in blood [11]. In a previous review, it is suggested that regulation of AdipoRs might be one potential target for therapy [11]. However, studies about the role of AdipoRs in colitis are still rare; only the study by Sinderi shows that short-term (2 days) AdipoR1 knock-down exacerbates colitis in mice [21]. The effects of long-term overexpression of AdipoR1 on colitis are still unknown.

To study the physiological role of AdipoR1 in intestinal inflammation, we employed DSS to induce murine colitis in porcine AdipoR1 transgenic mice (pAdipoR1 mice). We also used recombinant ADN protein to investigate the role of ADN-AdipoR1 signaling in colitis in the THP-1 macrophage cell line and the HT-29 human colon epithelial cell line.

## Methods

### Experimental animals

pAdipoR1 mice were generated as previously reported [33]. Both wild type (WT) and transgenic mice were maintained under the same breeding conditions, and with the same diet fed ad libitum to ensure similar intestinal microflora. We used eight-week old pAdipoR1 female mice constructed on a genetic background of FVB/N. Eight week old female FVB/N WT mice were used for the control group.

### Survival study

pAdipoR1 and WT mice (*n* = 3 for each group) were given different doses of dextran sodium sulfate (DSS) (0.5, 1, or 2%) (MW = 36,000–50,000, MP Biomedicals, Illkirch, France) in drinking water to induced colitis. We observed the mice for diarrhea and sluggish behavior and recorded body weight and food intake every day. The 2% DSS group mice were euthanized with CO_2_ on the ninth day and the 0.5 and 1% DSS group on the 15th day.

### Establishment of acute colitis

WT and pAdipoR1 mice (*n* = 8 for each group) were given 2% DSS in drinking water for 7 consecutive days to induce acute colitis and were euthanized with CO_2_ at the end of experiment. The body weight and clinical phenotypes as diarrhea, sluggish behavior and food intake were recorded daily. All the animal experiments were approved by the Institutional Animal Care and Use Committee (IACUC) at National Taiwan University.

### Histologic analysis

To evaluate the severity of colitis, the whole large intestine was harvested, fixed in 10% formalin for 15 h, then, embedded in paraffin. Paraffin sections (4 μm) were stained with hematoxylin and eosin. The sections were also reacted with various antibodies, AdipoR1 (5512–1; Epitomics, Burlingame, CA, United States), CD68 (ab125212; abcam, Cambridge, United Kingdom), cyclooxygenase/cox2 (SP21; Spring Bioscience, Inc., Pleasanton, CA, United states) and neutrophil elastase (bs-6982R; Bioss, Inc.). The endogenous peroxidase activity and non-specific binding were blocked by 0.3% H_2_O_2_/ methanol and 10% calf serum/PBS. The signal was detected by a horseradish peroxidase (HRP)/ diaminobenzidine (DAB) system according to the manufacturer’s instructions (Dako, Carpinteria, CA, United States).

### Protein extraction and western blotting analysis

The colon of WT and pAdipoR1 mice was homogenized in liquid nitrogen and total protein was extracted using the RIPA buffer (Cell Signaling, Beverly, MA, United States). Proteins were mixed with SDS-PAGE sample buffer and boiled for 5 mins. Proteins were separated by 10% SDS-PAGE and transferred to PVDF membranes. Membranes were blocked with 5% nonfat milk for 1 h at room temperature and incubated with antibodies against ADN (EPR3218, 1:1000, Epitomics, Burlingame, CA, United State) or β-actin (sc-47,778, 1:5000, Santa Cruz Biotechnology, Dallas, TX, United State) overnight at 4 °C. After washing with Tris buffered saline with Tween20, membranes were incubated with HRP-conjugated secondary IgG antibody (1: 5000, Cell signaling, Beverly, MA, United State) and the signal was detected using the ECL Western blotting substrate (Thermo Scientific, Rockford, IL).

### Colon tissue culture and enzyme-linked immunosorbent assay

After euthanasia of the mice, 0.2 g of the terminal colon was cut into pieces and soaked in 3 mL RPMI-1640 medium (SH300027; GE Healthcare Life Sciences, Marlborough, MA, United States) for 15 h, then centrifuged at 12,000 X g for 10 mins. The supernate was collected. The concentrations of IL-6 and TNF-α in the supernate were determined using mouse-specific enzyme-linked immunosorbent assay (ELISA) kits (eBioseience, San Diego, CA, United States). The serum ADN, IL-6 and TNF-α levels were quantified using mouse ADN, IL-6 and TNF-α ELISA kits (R&D System, Inc., Minneapolis, MN, United States).

### Cell culture

The human colon epithelial cell line, HT-29 (Food Industry Research and Development Institute, Hsinchu, Taiwan), was cultured in DMEM medium (SH30022; GE Healthcare Life Sciences) with 10% fetal bovine serum (FBS; Thermo Fisher Scientific, Waltham, MA, United States) and 1% glutamine (GE Healthcare Life Sciences). The human monocyte cell line, THP-1 (Food Industry Research and Development Institute), was cultured in RPMI-1640 medium (SH300027; GE Healthcare Life Sciences) with 10% FBS, 1% glutamine and 0.05 mM 2-mercaptoethanol. 2 × 10^6^ cells were plated on a 100 mm plate and cultured in humidified 95% air plus 5% CO_2_ at 37 °C. Cells were subcultured every three days.

### Adiponectin recombinant protein treatment

Full-length human adiponectin recombinant protein (ADN; mimics serum adiponectin by forming high molecular weight and hexameric species) was purchased from ENZO Life Sciences (ALX-522-063; ENZO Life Science, Farmingdale, NY, United States). After 6 h of serum deprivation, 1 μg/mL lipopolysaccharide (LPS; Sigma-Aldrich, St. Louis, MO, United States) or 5 μg/mL ADN were added to 5 × 10^5^ HT-29 cells for 18 h. 4 × 10^5^ suspended THP-1 cells were differentiated into plated macrophages by treating with 100 nM phorbol myristate acetate (PMA; Sigma-Aldrich) for 72 h. We added 1 μg/mL LPS or 5 μg/mL ADN for 18 h after 6 h’ serum starvation. Cells were harvested for RNA extraction and the culture media (conditioned medium) was collected for ELISA. The prostaglandin E2/PGE2 (ENZO Life Sciences), IL-8, TNF-α, IL-1 and IL-6 (eBioscience) expressions in conditioned media were detected using ELISA kits, as indicated above.

### RNA extraction, cDNA synthesis and reverse transcription-quantitative polymerase chain reaction (qPCR)

Total RNA was extracted with TRIzol reagent (Thermo Fisher Scientific, Inc.) and cDNA was synthesized using a high capacity cDNA reverse transcription kit (Applied Biosystems, Foster, CA, United States). qPCRs were performed with the DyNAmo Flash SYBR Green qPCR Kit (Thermo Fisher Scientific, Inc.) using a C1000™ Thermal Cycler (Bio-Rad, Hercules, CA, United States). qPCR was performed with an initial denaturation at 95 °C for 5 mins followed by 40 cycles at 95 °C for 30 s, then 60 °C for 60 s and 72 °C for 30 s; terminal extension was at 72 °C for 7 mins. The primers were listed in Additional file 1: Table S1.

### Immunohistochemistry quantification

The immunohistochemical signal (brown color) score was quantified by image J (https://imagej.nih.gov/ij/download.html).

### Statistical analysis

Data were expressed as mean ± SEM. A paired t test or one-way ANOVA followed by Tukey’s multiple comparison test were used for comparisons among groups. Means indicated by different letters were different at *P* ≤ 0.05.

## Results

### The expression of adiponectin and adiponectin receptor 1

**Fig. 1:**
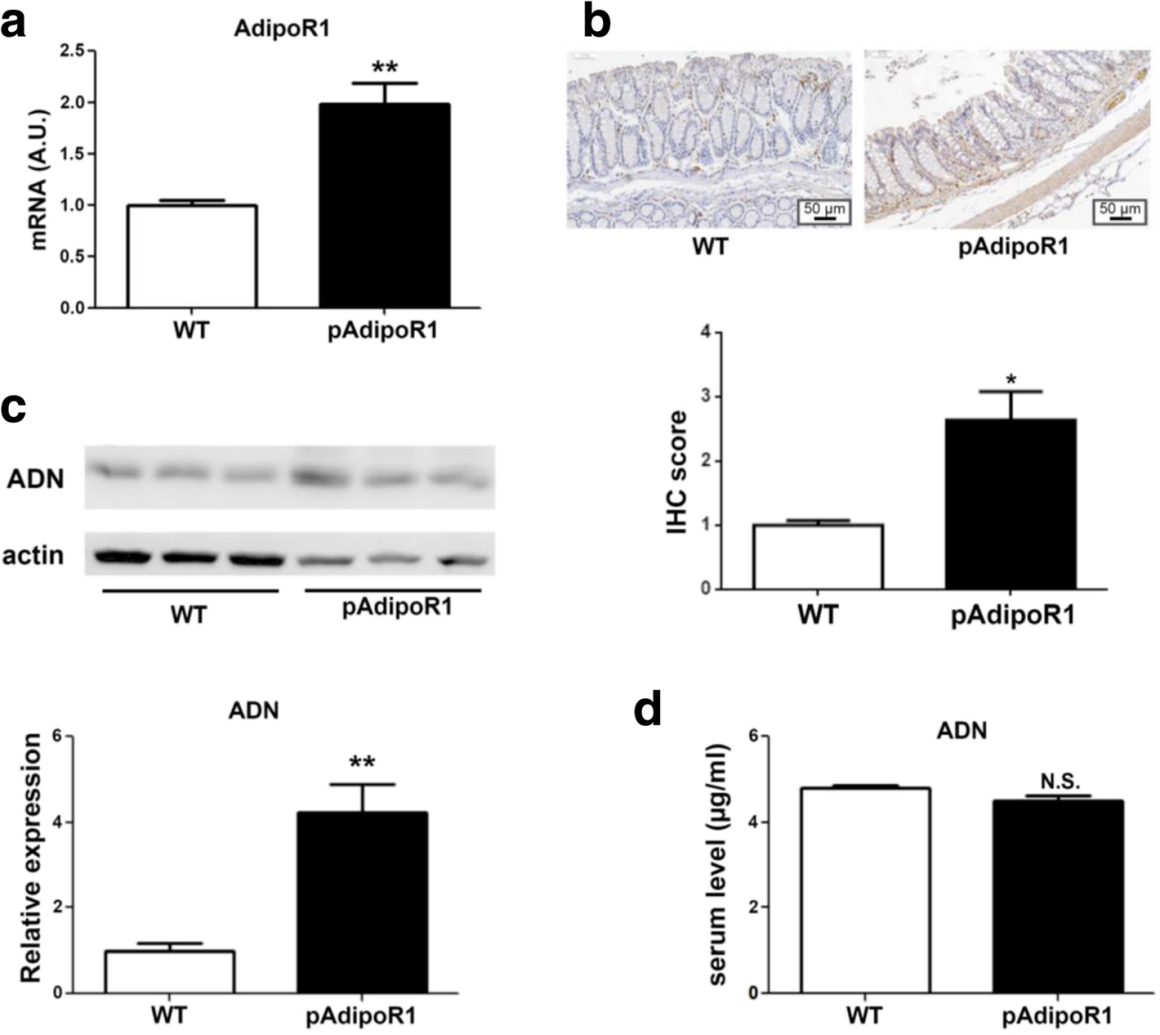
The expression of adiponectin and adiponectin receptor 1 in wild type (WT) and porcine adiponectin receptor 1 transgenic mice (pAdipoR1). **a** The AdipoR1 mRNA expression in the colonic tissue was detected by the quantitative polymerase chain reaction (qPCR). **b** Immunohistochemical staining (IHC) for AdipoR1 in mouse colon sections. **c** The adiponectin (ADN) protein expression was detected by western blot in the colonic tissue. **d** Enzyme-linked immunosorbent assay (ELISA) analysis of the serum level of ADN. Data in Fig. A to D were analyzed by t test (*n* = 6). N.S. means no-significant difference. * means *p* ≤ 0.05, ** means *p* ≤ 0.005

We used the chicken β-actin promoter to constantly drive the expression of pAdipoR1 in transgenic mice. Both mRNA and protein levels of AdipoR1 were overexpressed in the colon of pAdipoR1 mice (Fig. 1a and b). Moreover, the colonic ADN protein of pAdipoR1 mice was greater than in WT mice (Fig. 1c). However, the circulating level of ADN was not different between WT and pAdipoR1 mice (Fig. 1d).

### DSS-induced colitis

**Fig. 2:**
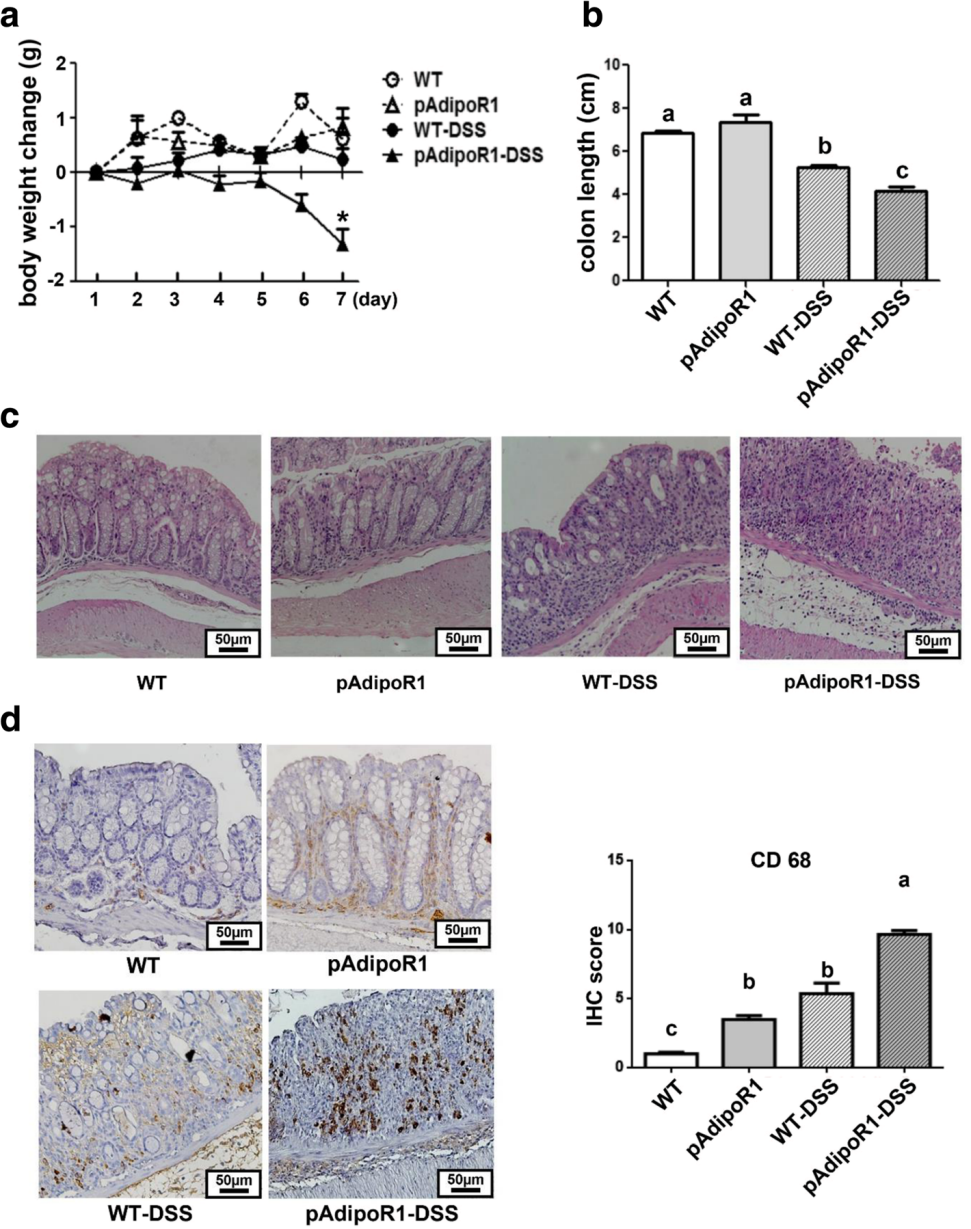
The responses of wild type mice (WT) and porcine adiponectin receptor 1 transgenic mice (pAdipoR1) to DSS-induced colitis. After 7 days of 2% dextran sodium sulfate (DSS) treatment, pAdipor1 mice had more severe colitis than WT mice. **a** body weight change (**b**) colon length (**c**) The mouse colon section was stained with hematoxylin and eosin. **d** Macrophage infiltration was recognized by CD68 detection (the brown color). Data in Fig. A were analyzed by two way ANOVA. * means *p* ≤ 0.05. Data in Fig. B and D were analyzed by one way ANOVA with mean separation using Tukey’s test. Means with different letters indicated *p* ≤ 0.05

To study the role of AdipoR1-overexpression in DSS-induced murine colitis, we assessed colitis symptoms by body weight loss, bloody-diarrhea, colon-length and histological analysis. Three independent experiments showed that pAdipoR1 mice developed more severe acute colitis than WT mice. After DSS induced murine colitis, pAdipoR1 mice had lower body weight from the seventh day (Fig. 2a) along with diarrhea, whereas these symptoms in WT mice were minimal. Furthermore, the colon-length of DSS-treated mice was shortened; pAdipoR1 mice were severely affected (Fig. 2b). In H & E stained sections, crypts from WT and pAdipoR1 mice were indistinguishable. However, there was greater crypt injury in the pAdipoR1-DSS mice than in WT-DSS mice (Fig. 2c).

### Macrophage infiltration

To examine the macrophage infiltration in the colonic tissue of mice, we detected the expression of the macrophage marker, CD68 by immunohistochemistry. There was greater macrophage infiltration in pAdipoR1 than in WT colon tissue (Fig. 2d). After 7 days of DSS induced murine acute colitis, the expression of CD68 was greater in the colon of pAdipoR1-DSS mice than in the colon of WT-DSS mice (Fig. 2d).

### IL-6 and TNF-α in mouse serum and colonic tissue culture medium

**Fig. 3:**
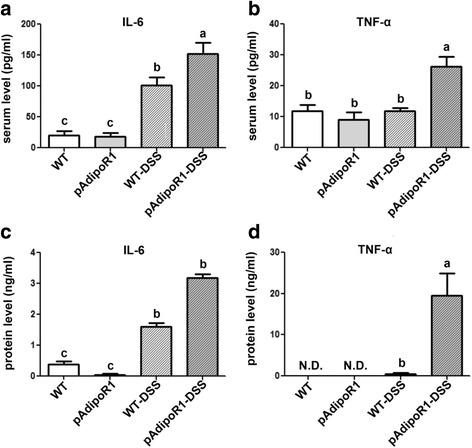
The expression of pro-inflammatory cytokines in serum and colon of wild type mice (WT) and porcine adiponectin receptor 1 transgenic mice (pAdipoR1). **a** and **b** The expression of IL-6 and TNF-α in the serum of mice. **c** and **d** The IL-6 and TNF-α expression and secretion to the medium in the murine colon culture supernates. Data in Fig. A to D were analyzed by one-way ANOVA with mean separation using Tukey’s test. Means with different letters indicated *p* ≤ 0.05. N.D. means not detectable

IL-6 and TNF-α are important acute pro-inflammatory cytokines secreted by macrophages. IL-6 and TNF-α levels were low in the serum (Fig. 3a and b) and culture medium (Fig. 3c and d) from the colons of pAdipoR1 and WT mice. WT mice with acute colitis had increased serum levels of IL-6 compared to WT mice, but the serum level of TNF-α was not different. The serum IL-6 and TNF-α levels were greater in pAdipoR1-DSS mice with colitis (Fig. 3a and b). Furthermore, the IL-6 concentration in the medium from cultured colon was increased after DSS treatment and was even greater in medium from pAdipoR1-DSS mice colon (Fig. 3c). The TNF-α concentration was not increased in medium from cultured WT-DSS colons, but was markedly increased in medium from cultured pAdioR1-DSS colons (Fig. 3d). Therefore, both the circulating and colonic cell medium levels of IL-6 and TNF-α were higher in pAdipoR1 mice than in WT mice after acute colitis.

### The effect of adiponectin on the cyclooxygenase/prostaglandin E2 pathway

**Fig. 4:**
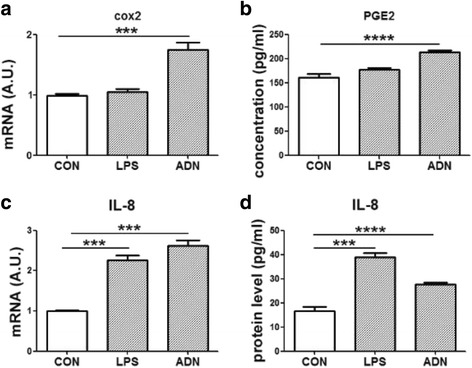
The expression of inflammatory markers in a colon epithelial cell line (HT-29 cells). HT-29 cells were treated with 1 μg/mL lipopolysaccharides (LPS) or 5 μg/mL full length adiponectin (ADN) for 18 h. **a** the relative mRNA expression of cox2. **b** the PGE2 secretion to the culture medium. **c** the relative mRNA expression of IL-8. **d** the IL-8 secretion to the culture medium. Data were analyzed by one-way ANOVA with mean separation using Tukey’s test. Each bar represents the mean ± SEM; a value of *p* ≤ 0.05 was considered significant. *** means *p* ≤ 0.0005, **** means *p* ≤ 0.0001

**Fig. 5:**
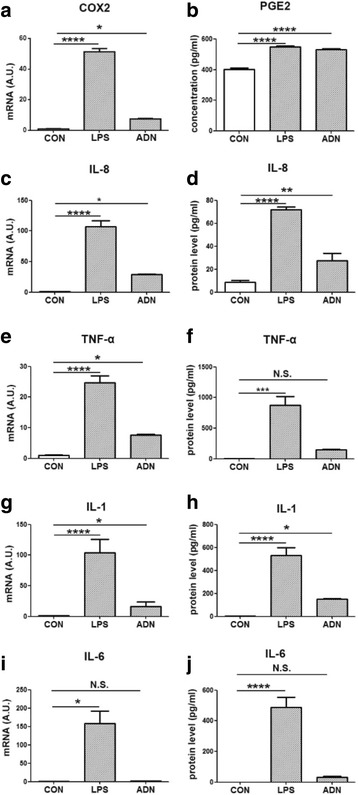
The expression of inflammatory markers in macrophages. THP-1 cells were treated with1 μg/mL lipopolysaccharides (LPS) or 5 μg/mL full length adiponectin (ADN) for 18 h. **a** the relative mRNA expression of cox2. **b** the PGE2 secretion to the culture medium. **c** the relative mRNA expression of IL-8. **d** the IL-8 secretion to the culture medium. **e** the relative mRNA expression of TNF-α. **f** the TNF-α secretion to the culture medium. **g** the relative mRNA expression of IL-1. **h** the IL-1 secretion to the culture medium. **i** the relative mRNA expression of IL-6. **j** the IL-6 secretion to the culture medium. Data were analyzed by one-way ANOVA with mean separation using Tukey’s test. Each bar represents the mean ± SEM; a value of *p* ≤ 0.05 was considered significant. * means *p* ≤ 0.05, ** means *p* ≤ 0.005, *** means *p* ≤ 0.0005, **** means *p* ≤ 0.0001

Upregulation of cox2 and PGE2 are important in promoting acute inflammation [34]. In human colon epithelial HT-29 cells, addition of AND but not LPS enhanced cox2 mRNA expression and PGE2 secretion (Fig. 4a and b). In human macrophage THP-1 cells, both LPS and ADN increased the expression of cox2 and PGE2 (Fig. 5a and b). ADN enhanced the cox2-PGE2 pathway in colon epithelial and macrophage cell lines.

### Adiponectin enhanced the expression of IL-8

IL-8 is a chemokine inducing chemotaxis in target cells, primarily in neutrophils in humans [35]. In HT-29 and THP-1 cells, 18 h treatment with LPS or ADN enhanced mRNA expression and protein secretion of IL-8. The LPS treatment was more effective than the ADN treatment in the THP-1 cells (Fig. 4c and d; Fig. 5c and d).

### The effect of adiponectin on pro-inflammatory cytokines

TNF-α, IL-1 and IL-6 are important pro-inflammatory cytokines in acute inflammation. In our study, LPS or ADN did not affect the mRNA expression or protein secretion of TNF-α or IL-6 in HT-29 cells (data not indicated). In THP-1 cells, TNF-α, IL-1 and IL-6 were increased by the inflammatory inducer, LPS. Like LPS, ADN treatment enhanced the level of TNF-α and IL-1 but not IL-6 (Fig. 5e-j).

### Adiponectin receptor 1 enhanced the expression of neutrophil chemokines, cox2 and elastase in mice colon tissue

**Fig. 6:**
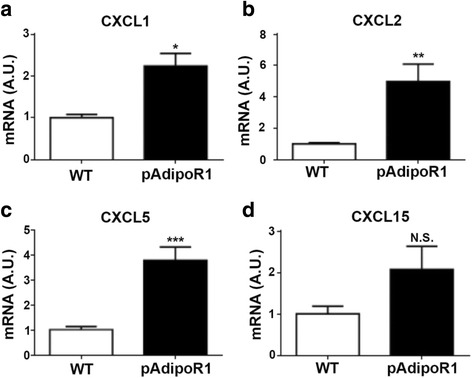
The expression of neutrophil chemokines in the colon of mice. **a** CXCL1 **b** CXCL2 (**c**) CXCL5 **d** CXCL15 Data in Fig. A to D were analyzed by Student’s t test (*n* = 6). N.S. means no-significant difference. * means *p* ≤ 0.05, ** means *p* ≤ 0.005, *** means *p* ≤ 0.0005

**Fig. 7:**
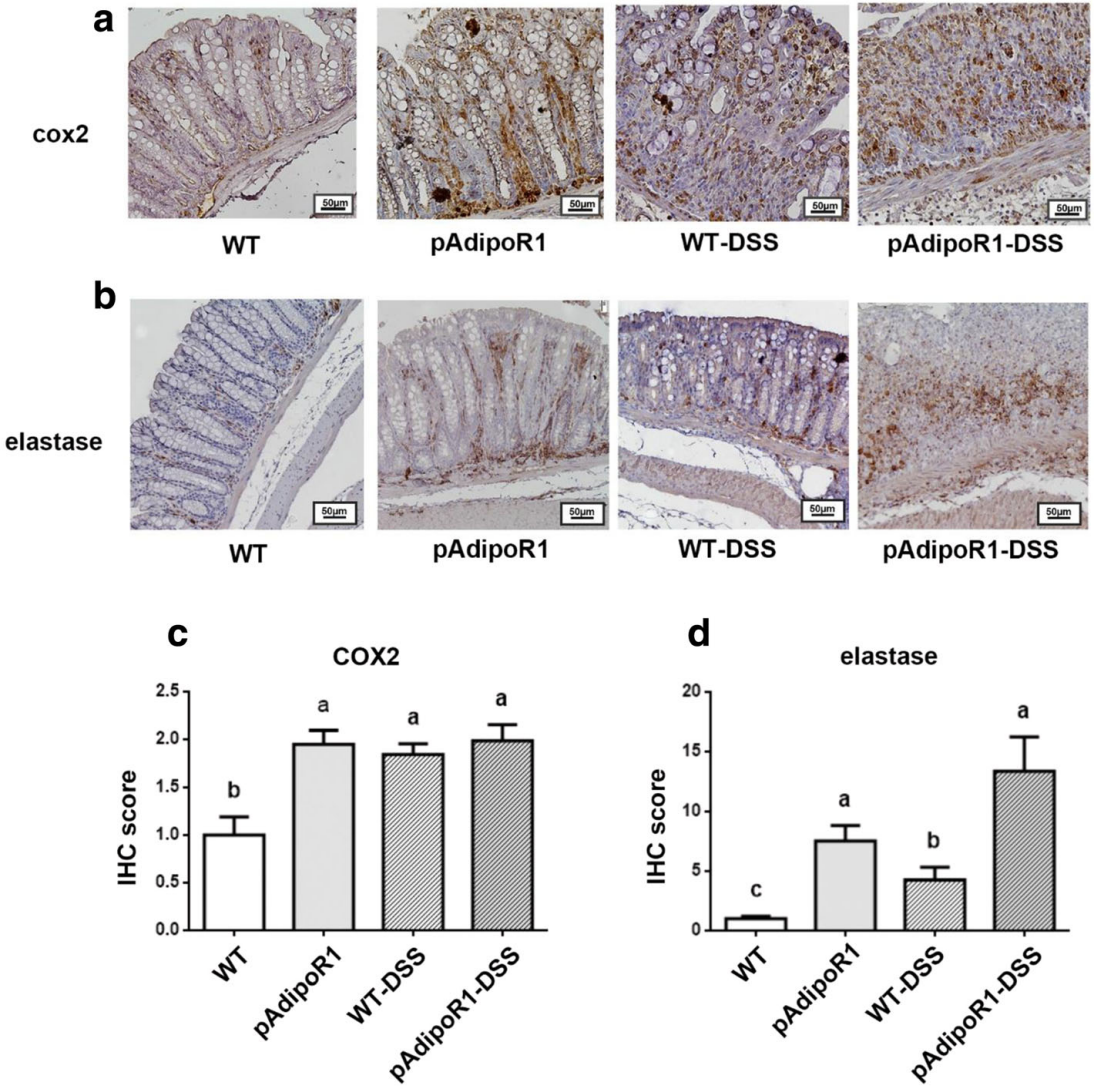
The expression of pro-inflammatory factors and neutrophils in the colon of mice. **a** cox2 **b** elastase **c** Quantitative analysis of cox2-positive area (**d**) Quantitative analysis of elastase-positive area. Neutrophil infiltration was recognized by elastase detection (the brown color). Data were analyzed by one way ANOVA with mean separation using Tukey’s test. Means with different letters indicated *p* ≤ 0.05. IHC score: immunohistochemical signal score

We observed that pAdipoR1-overexpression elicited murine colitis and ADN recombinant protein enhanced the expression of the pro-inflammatory factors IL-8 and cox2-PGE2 in colon epithelial cells (HT-29). As mice lack IL-8, we detected the expression of CXCL1, CXCL2, CXCL5 and CXCL15, to estimate similar neutrophil recruitment and function. The expression of CXCL1, CXCL2 and CXCL5 were significantly higher in the colon of pAdipoR1 than WT mice (Fig. 6a-d). Furthermore, in WT mice, colonic cox2 was rarely detected in the healthy colon, but was increased in inflamed colon. However, expression of colonic cox2 in mice without colitis was greater in pAdipoR1 than in WT mice (Fig. 7a).

Our study showed that ADN and AdipoR1 enhanced the neutrophil chemokines (IL-8, CXCL1, CXCL2 and CXCL5) in human cell lines or mice colon. To investigate whether the increasing chemokines affect neutrophil infiltration, we detected the expression of neutrophil elastase, a marker for neutrophils in mice colon. Neutrophil infiltration was rarely detected in WT mice, but could be detected in pAdipoR1 mice. In the colitis groups, there was considerably more elastase expressed in the WT-DSS than in control-WT mice. However, colitis did not further elevate the elastase levels in the pAdipoR1-DSS compared to pAdipoR1 mice (Fig. 7b).

In summary, AdipoR1 overexpression increased the expression of cox2 and neutrophil chemokines to promote neutrophil infiltration into the colon of pAdipoR1 mice. The excessive cox2 and neutrophil infiltration might worsen murine colitis.

## Discussion

Numerous studies demonstrate that ADN functions as an anti-inflammatory agent and participates in improvement of metabolic inflammatory diseases, such as type 2 diabetes, obesity, and cardiovascular diseases [36–38]. Low adiponectinemia has been observed in patients with metabolic disease [39–42]. Therefore, ADN is considered a potential therapeutic target for metabolic disease [15]. However, due to the rather great plasma concentration (2 to 20 μg/mL) of ADN and its multiple forms, the utility of ADN as a pharmacological agent is problematical [11]. There are only a few studies regarding the effects of AdipoR1 on metabolic disease, therefore, we examined the therapeutic potential of AdipoR1 in this study. Both ADN and AdipoR1 proteins are highly conserved between humans, pigs and mice. The pig ADN has 83 and 81% identity with the human and mouse, respectively. The pig AdipoR1 has about 97% identity with both human and mouse forms [43]. Moreover, previous study determined the crystal structure of human AdipoR1 and revealed its zinc-binding site and putative ADN-binding surface [44]. The binding of ADN to the C-terminal extracellular region/CTR (residues 365–375) of AdipoR1 and the zinc-binding site (residues 187–212, 333–347) of ADN may have a role in the ADN-stimulated phosphorylation of AMPK and the up-regulation of UCP2 [44]. The sequence alignment of human, pig and mouse AdipoR1 showed the CTR and zinc-binding site of mouse and pig AdipoR1 are identical (Additional file 2: Figure S1). Furthermore, previous studies showed that ADN binds to AdipoR1 to induce the phosphorylation of AMPK [45]. We isolated porcine adipocyte and used the ADN treated-porcine adipocyte to confirm whether the mouse ADN can activate porcine AdipoR1. The porcine adipocyte was treated with 10 μg/mL murine ADN recombination protein (ALX-522-059; ENZO Life Science, Farmingdale, NY, USA) and the phosphorylation levels of AMPK were detected at 0, 5 and 15 min. The result showed the mouse ADN increased the level of phosphorylated AMPK at 15 min in porcine adipocyte (Additional file 3: Figure S2). It implies that mouse ADN could activate mouse and pig AdipoR1.

Previously, we established the pAdipoR1 mice carrying the transgenic porcine AdipoR1 conjugated with flag-tag (Additional file 4: Figure S3A). To confirm whether the mouse ADN binds to porcine AdipoR1, we performed co-immunoprecipitation(IP) using anti-ADN antibody (ab22554; Abcam, Cambridge, MA, USA) and detected the porcine AdipoR1 using anti-flag antibody (F1804; Sigma-Aldrich, St. Louis, MO, USA) in the colon of WT and pAdipoR1 mice. Flag is detectable in the protein of pAdipoR1 mice performed ADN IP (Additional file 4: Figure S3B). It suggests that mouse ADN binds to porcine AdipoR1 in pAdipoR1 mice. Moreover, we reported that the overexpression of pAdipoR1 resists the weight gain, hepatosteatosis, insulin resistance and heart hypertrophy in mice fed a high-fat/sucrose diet [33, 46, 47]. These results suggest that up-regulation of AdipoR1 might be a potential strategy for therapy of metabolism-related diseases.

In earlier studies, the prevalence of IBD was not considered gender-related [6]. However, an extensive clinical study recently showed IBD was more prevalent in females compared with males [48]. To avoid the interference of the menstrual cycle, previous studies usually used male mice. In this study, we used a DSS-induced colitis in both male and female mice. Both genders of pAdipoR1 mice showed more severe colitis symptoms than WT mice (data not shown). However, the difference is more significant in female mice. Therefore, we are the first to use female mice to study AdipoR1 effects in colitis.

The role of ADN in clinical and animal studies of IBD is controversial. Some studies show that the level of ADN in serum and mesenteric adipose tissue is increased in IBD patients [49–51]. Others demonstrate that ADN is down-regulated in serum and adipose tissue in IBD patients [52]. However, some studies indicate that there is no correlation between IBD and the level of ADN in serum [53, 54]. For example, knockout of ADN suppresses chemically-induced colonic inflammation in mice [22, 23]; in contrast, the study by Nishihara shows that ADN displays protective effects against murine colitis [18].

Aberrant immunoregulation is a portion of the pathogenesis in IBD. The disturbance of pro- and anti-inflammatory cytokines and the infiltration of activated immune cells (e.g., T cells, B cells, natural killer cells, macrophages and neutrophils) into intestinal mucosa are involved in the aggravation of mucosal inflammation in IBD [25]. In clinical studies, the high level of IL-8 (an chemoattractant for neutrophils), CXCR1 (the IL-8 receptor), and the high amount of neutrophils in serum are positively correlated with inflammation in IBD patients [55–61]. ADN enhances the IL-8 signaling through AdipoR1, but not AdipoR2 in hepatocytes, [32]. Discontinued proliferation of plasma neutrophils coupled with the short lifespan of neutrophils, cause neutrophils to be present in the blood only a few days. Delayed apoptosis in neutrophils in IBD patients is relevant to pathological intestinal inflammation [62, 63]. Due to the effects of neutrophil chemotaxis and the delayed apoptosis in IBD, depletion of neutrophils and blockade of neutrophil adhesion may be an effective strategy for IBD therapy [27, 64, 65].

Previous studies demonstrate that ADN inhibits neutrophil apoptosis and enhances the expression of neutrophil-attracting chemokines (CXCL1 and CXCL2 in colonic cells; IL-8 in fibroblasts and hepatocytes) [22, 30–32]. In this study, we showed a requirement of AdipoR1 signaling in murine colitis through regulating of neutrophil chemotaxis. We observed the accumulation of neutrophils and the increased of CXCL1, CXCL2, and CXCL5 in the colon of pAdipoR1 mice. Moreover, ADN enhanced the expression and secretion of IL-8 in a human colonic epithelial and macrophage cell lines. We speculated that the activation of neutrophils could be the critical reason for the severe colitis phenotype in pAdipoR1 mice.

It is well known that the cox2/PGE2 pathway plays an important role in inflammation. Our data showed that ADN and AdipoR1 enhanced the expression of colonic cox2, like to Arsenescu’s previous report [17]. However, contrary to our results, the data by Arsenescu shows that although ADN induces the expression of cox2, it mediates a protective effect toward colitis and that cox2 knock-out mice are more susceptible to DSS-induced colitis [17, 66]. The serum levels of cox2 and PGE2 are increased in IBD patients, which may relate to the pro-inflammatory function of cox2/PGE2 pathway [67, 68]. Previous studies show that ADN is a biomarker for rheumatoid arthritis, and that ADN induces the expression of cox2 and PGE2 in rheumatoid arthritis synovial fibroblasts [69, 70]. Our results implied that the ADN-AdipoR1 signaling enhanced colonic inflammation like occurrences in rheumatoid arthritis.

Moreover, the long-term overexpression of AdipoR1 might result in an imbalance of the colonic immune system by increased neutrophil chemotaxis. Our findings strengthen the view of the multifaceted role of ADN-AdipoR1 signaling that must be considered carefully in disease therapy in the future. However, there are limitation to this study [17] The ADN-AdipoRs signaling is complicated, and ADN exists full-length and in globular forms in plasma and binds to AdipoR1 and AdipoR2. We only tested the effects of full-length recombinant ADN protein in cell models and AdipoR1 in a mouse model. We have not verified what kind of ADN induced colitis in mice and the role of AdipoR2 in IBD is still unclear. [48] We observed that ADN-AdipoR1 signaling increases neutrophil chemotaxis, but we do not know whether the inhibition of neutrophil infiltration can improve colitis.

## Conclusions

**Fig. 8:**
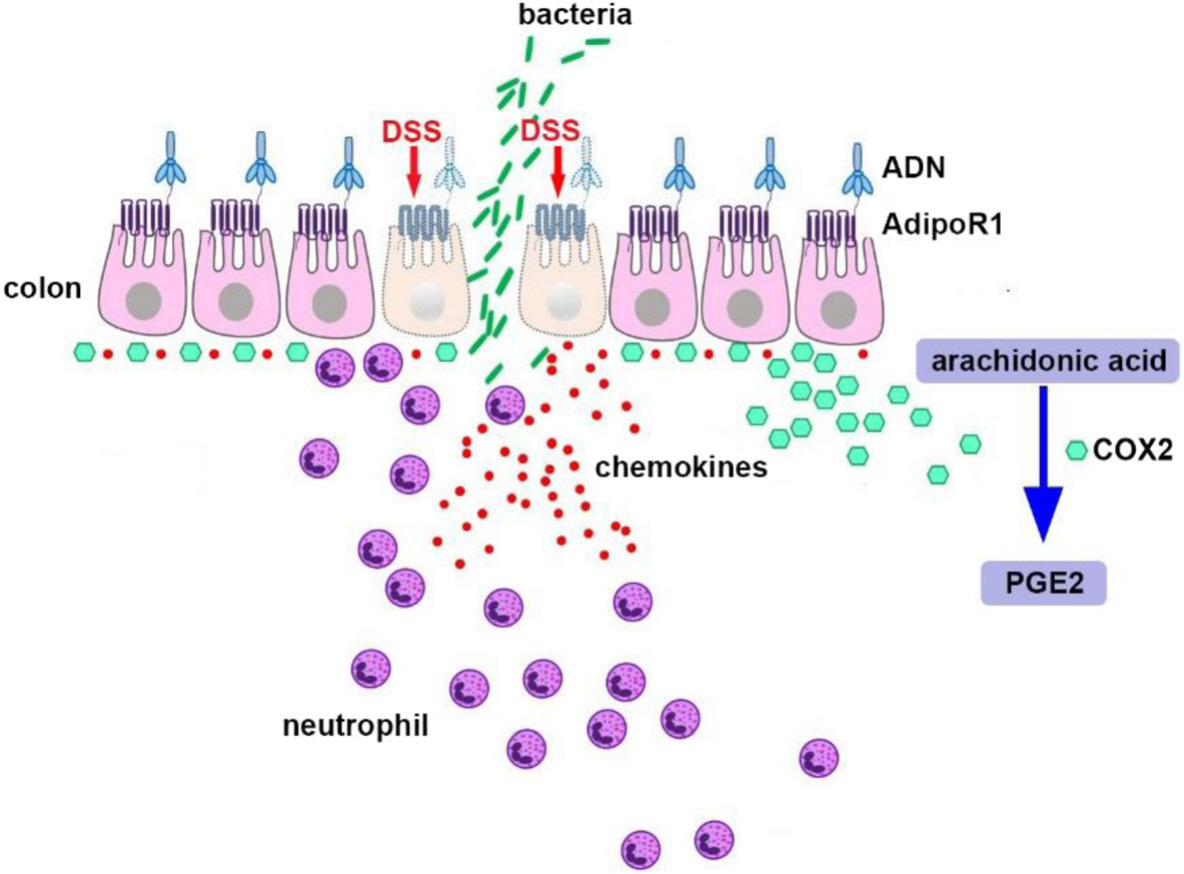
Proposed model of the ADN-AdipoR1 effect in colitis. ADN-AdipoR1 enhanced the neutrophil chemokines and cox2 production in colon epithelial cells. The excessive cox2 enhanced the expression of PGE2 and excessive neutrophil chemokines probably attract neutrophils into the colon and worsen DSS-induced colitis

In this study, the long-term overexpression of AdipoR1 exacerbated murine colitis induced by DSS and ADN enhanced the expression of pro-inflammatory factors in macrophage and colon epithelial cells. We proposed two possible reasons that ADN-AdipoR1 signaling exacerbated colonic inflammation in this study. ADN enhances the expression of cox2 promoting the production of PGE2 from arachidonic acid (AA) in the colon. Moreover, excessive neutrophil chemokines probably contribute to the attraction of neutrophils and enhance inflammation in the colon. Thus, ADN may be one of the major factors involved in colitis and IBD. In the pAdipoR1 mice, long-term overexpression of AdipoR1 might result in an imbalance of the immune system by neutrophil migration into the colon. Therefore, factors targeting ADN-AdipoR1 function may be useful candidates to improve colitis (Fig. 8).

## Additional files

Additional file 1: **Table S1.** Primer sets for quantitative real-time PCR. Primers were designed using the Primer-BLAST tool of National Center for Biotechnology Information (NCBI). Each primer sequence was confirmed by aligning its reference sequence in the NCBI database. (PDF 202 kb)

Additional file 2: **Figure S1.** Sequence alignment of human, pig and mouse AdipoR1. The sequence of *Homo sapiens*, *Sus scrofa*, and *Mus musculus* AdipoR1 were aligned using the software BioEdit. The sequences and their GenBank accession numbers are: *Homo sapiens* (NP_057083.2), *Sus scrofa* (NP_001007194) and *Mus musculus* (NP_082596.2). Boxes show the zinc-binding site (residues 187–212, 333–347) and C-terminal extracellular region/CTR (residues 365–375) of AdipoR1 which are conserved between pig and mouse. (PDF 216 kb)

Additional file 3: **Figure S2.** Mouse ADN recombinant protein induced the phosphorylation of AMPK in porcine adipocyte. (A) Porcine adipocyte was treated with mouse ADN recombination protein (10 μg/mL); the levels of AMPK, phospho-AMPK, and actin at 0, 5 and 15 min after treatment were detected by western blot. (B) Densitometric analysis of phospho-AMPK and AMPK levels was conducted using image J software. Mouse ADN increased the ratio of phosphor-AMPK and AMPK at 15 min in porcine adipocyte. Data were analyzed by one way ANOVA with mean separation using Tukey’s test. Means with different letters indicated *p* ≤ 0.05. (PDF 229 kb)

Additional file 4: **Figure S3.** Mouse ADN bound to porcine AdipoR1 in pAdipoR1 mice. (A) Mouse ADN (mADN) bound with mouse AdipoR1 in wild type (WT) mice, and mADN bound with mouse and flag-conjugated porcine AdipoR1 in pAdipoR1 mice. (B) Co-immunoprecipitation (co-IP) between m-ADN and flag-pAdipoR1. To confirm the mADN binds with pAdipoR1, IP was performed using anti-ADN antibody followed by immunoblotting using anti-flag antibody and anti-ADN antibody in the colon of WT and AdipoR1 mice. Flag-pAdpoR1 was detectable in the protein of AdipoR1 mice. ADN was detectable both in the WT and AdipoR1 mice. mADN: mouse adiponectin; mAdipoR1: mouse adiponectin receptor 1; f-pAdipoR1: porcine adiponectin receptor 1 conjugated with flag; IB: immunoblotting. (PDF 187 kb)
