# Supplementary material for: Adiponectin and adiponectin receptor 1 overexpression enhance inflammatory bowel disease
Source: J Biomed Sci. 2018 Mar 14;25:24. doi: 10.1186/s12929-018-0419-3 (PMC5851065; doi:10.1186/s12929-018-0419-3)
Supplement: Supplementary file 1 — Table S1. Primer sets for quantitative real-time PCR. Primers were designed using the Primer-BLAST tool of National Center for Biotechnology Information (NCBI). Each primer sequence was confirmed by aligning its reference sequence in the NCBI database. (PDF 202 kb) [file 12929_2018_419_MOESM1_ESM.pdf]

Supplementary Table 1. Primer sets for quantitative real-time PCR

	Primer sequence
mouse-AdipoR1 (NM_001306069)	5’ CCAGGAAGAAACCACCGGA 3’
	5’ GAAATCAGGAAGGCTGCCAAG 3’
mouse-β-actin (NM_007393)	5’ TGTTACCAACTGGGACGACA 3’
	5’ CTTTTCACGGTTGGCCTTAG 3’
mouse-CXCL1 (NM_008176.3)	5' GGTGTCCCCAAGTAACGGAG 3'
	5' TTGTCAGAAGCCAGCGTTCA 3'
mouse-CXCL2 (NM_009140)	5’ GCCCAGACAGAAGTCATAGCC 3’
	5’ TTCTTCCGTTGAGGGACAGC 3’
mouse-CXCL5 (NM_009141)	5' GCATCTAGCTGAAGCTGCC 3'
	5' CTATGACTTCCACCGTAGGGC 3'
mouse-CXCL15 (NM_0011339.2)	5' CTAGGCATCTTCGTCCGTCC 3'
	5' TTCACCCATGGAGCATCAGG 3'
human-cox2 (NM_000963)	5’ TGAGCATCTACGGTTTGCTG 3’
	5’ TGCTTGTCTGGAACAACTGC 3’
human-TNF-α (NM_000594)	5’ AGCCTCTTCTCCTTCCTGAT 3’
	5’ AAGATGATCTGACTGCCTGG 3’
human-IL-8 (NM_000584)	5’ CCAGGAAGAAACCACCGGA 3’
	5’ GAAATCAGGAAGGCTGCCAAG 3’
human-IL-6 (NM_000600)	5’ TGGAGATGTCTGAGGCTCATTC 3’
	5’ GAAGGCAACTGGACCGAAGG 3’
human-β-actin (NM_01101)	5’ GAAGATCAAGATCATTGCTCCT 3’
	5’ CTAAGTCATAGTCCGCCTAGAA 3’

Primers were designed using the Primer-BLAST tool of National Center for Biotechnology Information (NCBI). Each primer sequence was confirmed by aligning its reference sequence in the NCBI database.
