# Supplementary material for: Adiponectin and adiponectin receptor 1 overexpression enhance inflammatory bowel disease
Source: J Biomed Sci. 2018 Mar 14;25:24. doi: 10.1186/s12929-018-0419-3 (PMC5851065; doi:10.1186/s12929-018-0419-3)
Supplement: Supplementary file 2 — Figure S1. Sequence alignment of human, pig and mouse AdipoR1. The sequence of Homo sapiens, Sus scrofa, and Mus musculus AdipoR1 were aligned using the software BioEdit. The sequences and their GenBank accession numbers are: Homo sapiens (NP_057083.2), Sus scrofa (NP_001007194) and Mus musculus (NP_082596.2). Boxes show the zinc-binding site (residues 187–212, 333–347) and C-terminal extracellular region/CTR (residues 365–375) of AdipoR1 which are conserved between pig and mouse. (PDF 216 kb) [file 12929_2018_419_MOESM2_ESM.pdf]

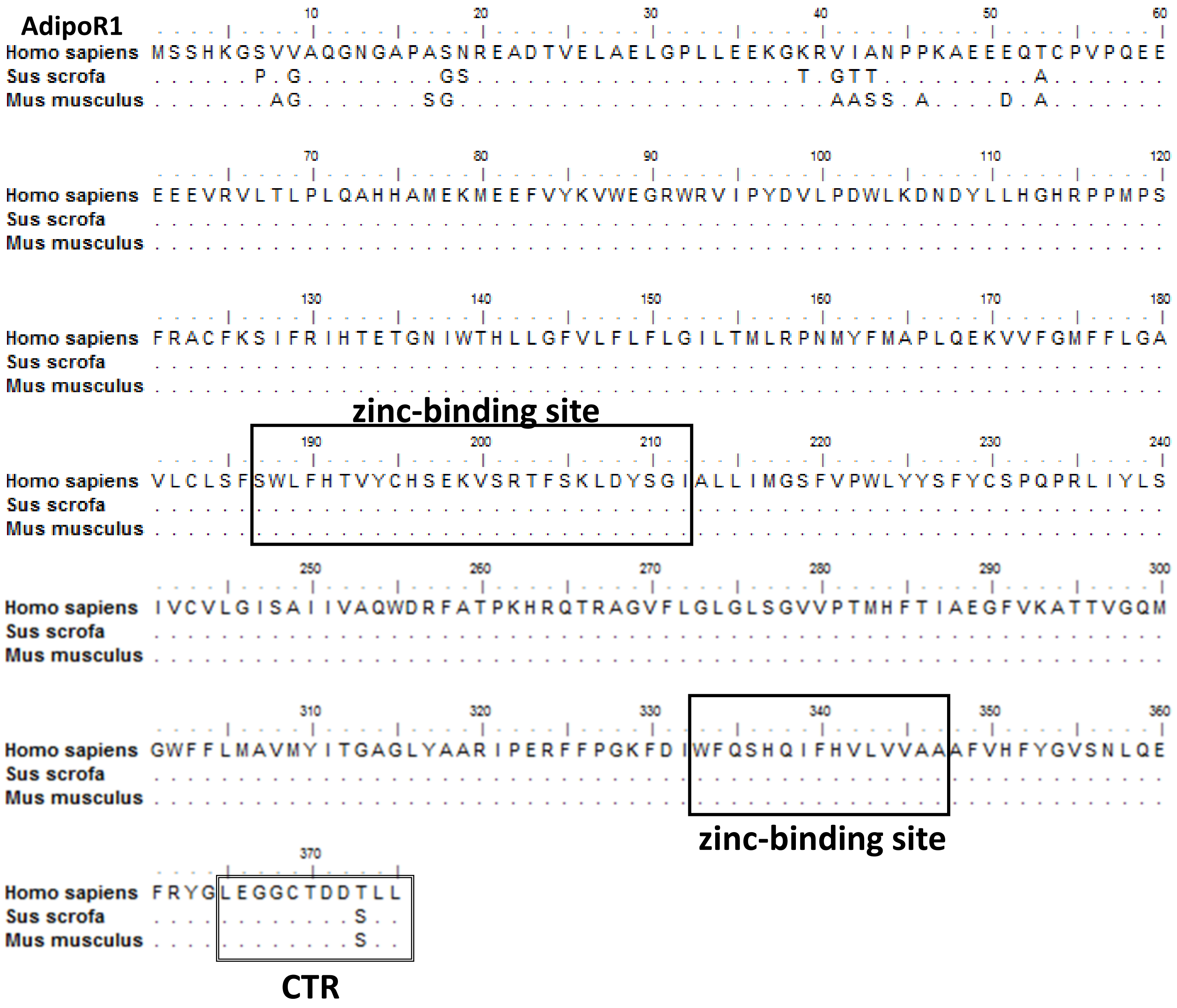

Supplementary Figure 1
