# Supplementary figures and images for: Adiponectin and adiponectin receptor 1 overexpression enhance inflammatory bowel disease
Source: J Biomed Sci. 2018 Mar 14;25:24. doi: 10.1186/s12929-018-0419-3 (PMC5851065; doi:10.1186/s12929-018-0419-3)

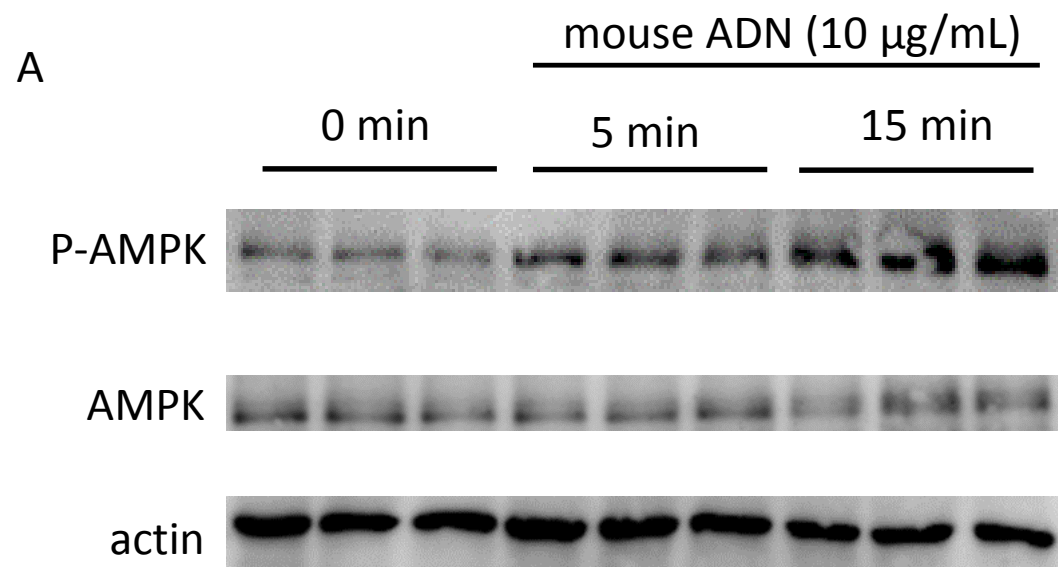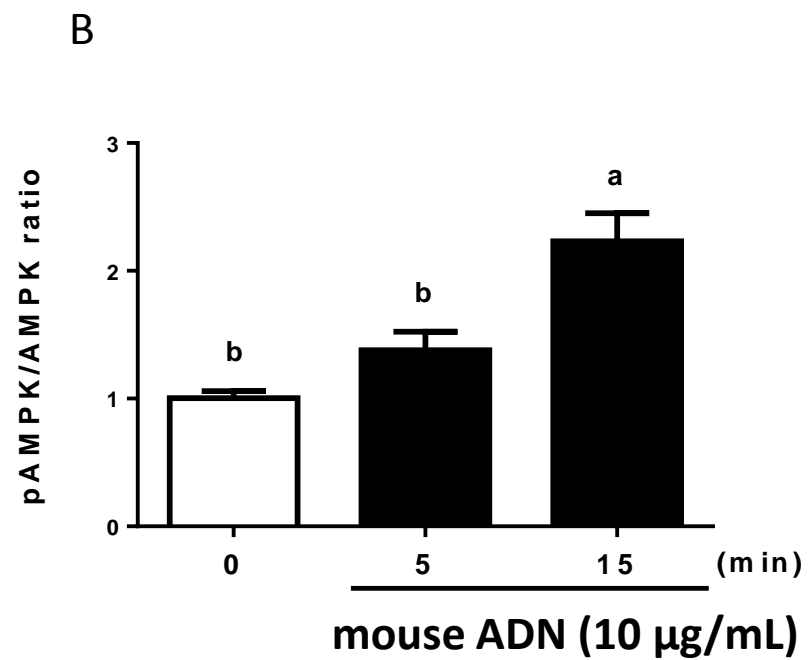

**Supplementary Figure 2**

Supplement: Supplementary file 3 — Figure S2. Mouse ADN recombinant protein induced the phosphorylation of AMPK in porcine adipocyte. (A) Porcine adipocyte was treated with mouse ADN recombination protein (10 μg/mL); the levels of AMPK, phospho-AMPK, and actin at 0, 5 and 15 min after treatment were detected by western blot. (B) Densitometric analysis of phospho-AMPK and AMPK levels was conducted using image J software. Mouse ADN increased the ratio of phosphor-AMPK and AMPK at 15 min in porcine adipocyte. Data were analyzed by one way ANOVA with mean separation using Tukey’s test. Means with different letters indicated p ≤ 0.05. (PDF 229 kb) [file 12929_2018_419_MOESM3_ESM.pdf]

**A**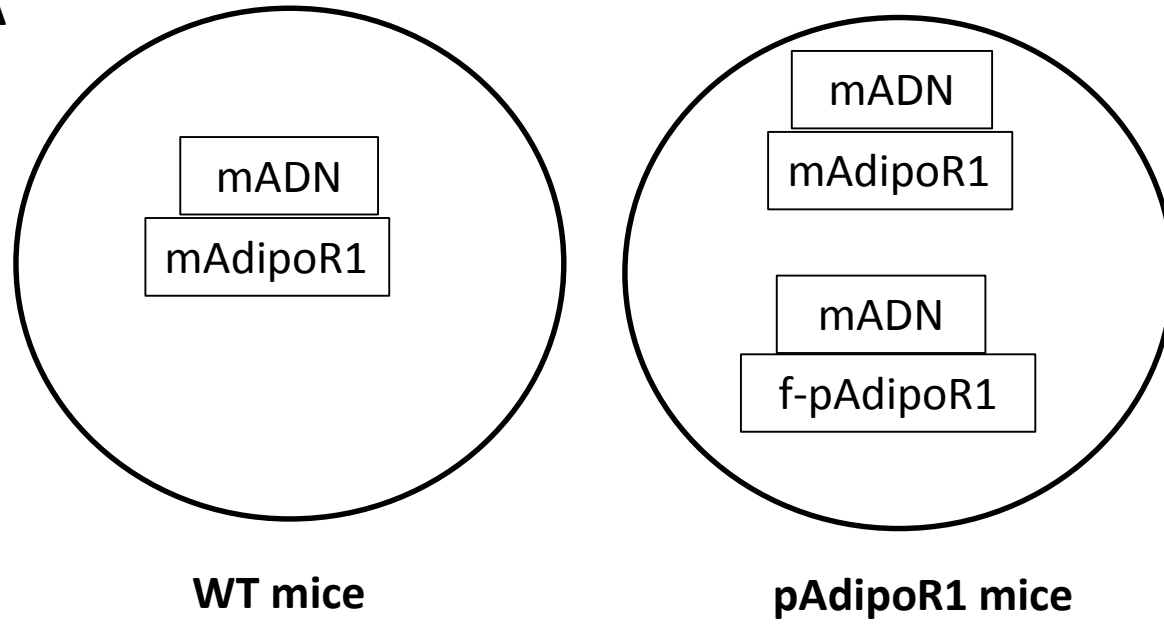**B**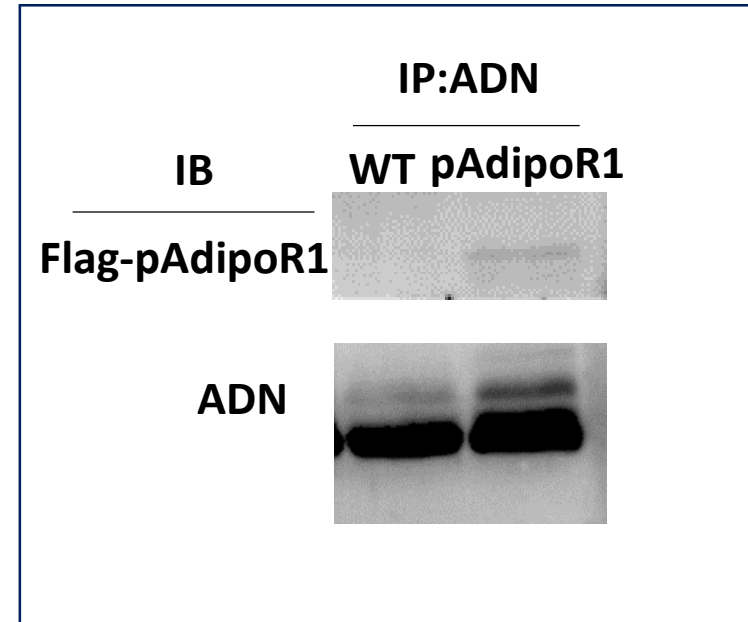

**Supplementary Figure 3**

Supplement: Supplementary file 4 — Figure S3. Mouse ADN bound to porcine AdipoR1 in pAdipoR1 mice. (A) Mouse ADN (mADN) bound with mouse AdipoR1 in wild type (WT) mice, and mADN bound with mouse and flag-conjugated porcine AdipoR1 in pAdipoR1 mice. (B) Co-immunoprecipitation (co-IP) between m-ADN and flag-pAdipoR1. To confirm the mADN binds with pAdipoR1, IP was performed using anti-ADN antibody followed by immunoblotting using anti-flag antibody and anti-ADN antibody in the colon of WT and AdipoR1 mice. Flag-pAdpoR1 was detectable in the protein of AdipoR1 mice. ADN was detectable both in the WT and AdipoR1 mice. mADN: mouse adiponectin; mAdipoR1: mouse adiponectin receptor 1; f-pAdipoR1: porcine adiponectin receptor 1 conjugated with flag; IB: immunoblotting. (PDF 187 kb) [file 12929_2018_419_MOESM4_ESM.pdf]
